# Clinical parameters associated with mortality in elderly patients with COVID-19

**DOI:** 10.1371/journal.pone.0332800

**Published:** 2025-09-23

**Authors:** Mehmet Gökhan Kaya, Kıvanç Karaman

**Affiliations:** 1 Emergency Medicine Service, Mugla Training and Research Hospital, Mugla, Turkey; 2 Department of Emergency Medicine, Faculty of Medicine, Mugla Sitki Koçman University, Mugla, Turkey; Ege University, Faculty of Medicine, TÜRKIYE

## Abstract

**Background:**

Elderly patients with COVID-19 face a heightened risk of severe outcomes and mortality, emphasizing the need for reliable early risk stratification in emergency departments (EDs). Clinical parameters such as respiratory rate, oxygen saturation, and peripheral perfusion index (PPI) have shown promise as simple and effective markers associated with mortality.

**Methods:**

This retrospective, single-center study included 144 COVID-19-positive patients aged 65 years and older admitted to a university hospital in Mugla, Turkey, from February 15, 2021, to April 15, 2023. Vital signs, shock indices, and PPI were collected from medical records. Receiver Operating Characteristic (ROC) curve analysis and binary logistic regression analysis were used to evaluate the associations of these parameters with in-hospital mortality.

**Results:**

Among 144 patients, 37 (26%) died during hospitalization. PPI ≤ 2.20 was the most sensitive parameter (area under the curve [AUC] = 0.684, sensitivity = 75.7%, negative predictive value [NPV] = 87.7%), allowing the early identification of patients at lower mortality risk. Oxygen saturation ≤ 86% demonstrated the highest specificity (79.4%) and positive predictive value [PPV] (46.3%), effectively identifying patients at high risk. Respiratory rate ≥ 25 breaths/min (AUC = 0.652) also showed significant association with mortality. Binary logistic regression analysis revealed that low PPI levels were independently associated with increased in-hospital mortality (Odds ratio [OR] = 4.067; 95% confidence interval [CI]: 1.668–9.913; p = 0.002).

**Conclusions:**

This study highlights the powerful association of readily available clinical parameters with mortality among elderly COVID-19 patients. PPI, in combination with respiratory rate and oxygen saturation, offers a cost-effective and efficient approach to enhance ED triage protocols. Incorporating these parameters into routine assessment could improve early identification of high-risk patients, optimize resource allocation, and save lives. Further prospective studies are warranted to validate these findings and advance risk stratification frameworks for acute care settings. Our findings should be considered as associations with mortality rather than strong predictive evidence.

## 1. Introduction

The COVID-19 pandemic has profoundly affected healthcare systems worldwide, with elderly patients being among the highest-risk groups [1]. Advanced age serves as a major risk factor for severe disease progression and is strongly associated with increased mortality in COVID-19 patients [2]. Early identification of high-risk elderly patients in the emergency department (ED) is crucial for timely and effective management. This necessitates the use of reliable and easily measurable clinical parameters.

Vital parameters such as blood pressure, pulse rate, body temperature, peripheral perfusion index (PPI), oxygen saturation, and respiratory rate are standard methods for assessing the physiological condition of patients upon ED admission. Moreover, indices like the shock index (SI) and modified shock index (MSI) have been shown to be associated with increased mortality and poor outcomes, particularly in elderly patients with comorbidities [3].

In addition to traditional vital parameters and their derived indices, PPI—an objective indicator of peripheral perfusion—has increasingly been recognized as a valuable tool for assessing disease outcomes, including prognosis. By analyzing the ratio between pulsatile and non-pulsatile components of blood flow, PPI provides insights into physiological processes inluenced by cardiac output and the dynamic interaction of the autonomic nervous system (sympathetic and parasympathetic). Understanding the role of these parameters may guide clinical decision-making and aid in prioritizing care for patients at higher risk of mortality [4,5].

This study aims to evaluate the significance of PPI, vital signs and shock indices in relation to mortality among COVID-19-positive patients aged 65 years and older presenting to the ED. By focusing on easily obtainable clinical data, it seeks to enhance patient outcomes through early risk assessment and targeted interventions.

## 2. Methods

### 2.1. Study design and settings

This retrospective, single-center, cross-sectional, observational study was conducted in the ED of a university-affiliated training and research hospital in Mugla, Turkey. The hospital, with a capacity of more than 600 beds, accommodates approximately 90,000 adult ED visits annually. Approval for the study was granted by the local ethics committee (Reference No: 240133-140). The research was conducted in accordance with the principles of the Declaration of Helsinki (1975, amended in 1983) and adhered to the Strengthening the Reporting of Observational Studies in Epidemiology (STROBE) guidelines (http://www.strobe-statement.org). The date of data access for research purposes was on December 1, 2024. During the research process, the authors did not have access to any information that could identify individual participants. All identifying data were kept strictly confidential and were not shared under any circumstances.

Due to the retrospective nature of the study, the requirement for written informed consent was waived.

### 2.2. Patient selection

From February 15, 2021, to April 15, 2023, the study included all patients aged 65 and older who were admitted to the ED and subsequently hospitalized with a laboratory-confirmed diagnosis of COVID-19, verified by a positive reverse transcriptase-polymerase chain reaction (RT-PCR) test. To ensure the reliability of the study, certain groups were excluded. These exclusions encompassed patients under 65 years of age, those with a history of peripheral artery disease, patients who chose to left the hospital against medical advice, had incomplete medical records, or were transferred to other healthcare facilities. Additionally, patients requiring emergency interventional procedures or diagnosed with specific conditions (e.g., acute coronary syndrome, cerebrovascular disease) were excluded, as their hospital admissions were unrelated to COVID-19. This applied even if incidental positive COVID-19 test results were identified. According to the literature, the mortality rate in COVID-19 patients over the age of 65 is reported to be approximately 40% [6]. Based on a *G*Power analysis assuming that 40% of patients were expected to have a fatal outcome, it was calculated that at least 124 patients would be required for the study to achieve 80% power with an effect size of 0.5. This minimum sample size was achieved; however, the study population remained relatively limited, which should be taken into account when interpreting the findings.

### 2.3. Data collection

The clinical data encompassed demographic characteristics (age and gender), vital signs (systolic and diastolic blood pressure, heart rate, respiratory rate, body temperature, oxygen saturation and PPI), calculated indices such as the Age-Adjusted Shock Index (AASI) and the Modified Shock Index (MSI), all retrieved from medical records. AASI was calculated by multiplying the traditional shock index (Heart Rate/ Systolic Blood Pressure) by the patient’s age (AASI = Shock Index × Age). MSI was calculated by dividing the heart rate by the mean arterial pressure (MSI = Heart Rate/ MAP). MAP was determined using the standard formula: (2 × Diastolic Blood Pressure + Systolic Blood Pressure)/ 3. Both indices were utilized in the analyses to support the objective assessment of the patients’ clinical status.

During the study period, all patients presenting to the ED of Mugla Training and Research Hospital with symptoms of COVID-19 infection were evaluated in the COVID-19 area of the ED. A trained triage nurse, who was continuously available in the ED, performed vital sign and PPI measurements for these patients. PPI values were obtained using the Philips G30E patient monitor system, which calculates the ratio between pulsatile and non-pulsatile components of the light detected by the pulse oximetry probe. The probe was placed on the index fingertip of the right hand, and the measurement was recorded once the value stabilized on the monitor or after 30 seconds. The researchers reviewed the records of patients aged 65 years and older who presented to the ED within the specified period and were diagnosed with COVID-19.

### 2.4. Outcome measures

The primary endpoint of this study was to identify the factors associated with mortality among patients aged 65 years and older who were admitted to the hospital with a laboratory-confirmed diagnosis of COVID-19 from the ED.

### 2.5. Statistical analysis

Data analysis was conducted using the IBM SPSS Statistics version 25.0 (SPSS Inc., Chicago, IL). The Kolmogorov–Smirnov test was used to assess the normality of data distribution. Since the data were not normally distributed, all quantitative variables were expressed as medians (minimum–maximum). Categorical variables were presented as frequencies (n) and percentages (%). Group comparisons were performed using the Mann–Whitney U test for continuous variables and either the chi-square test or Fisher’s exact test for categorical variables. Receiver Operating Characteristic (ROC) curve analysis was conducted to evaluate the diagnostic performance of parameters that showed significant differences for in-hospital mortality. Using Youden’s index (sensitivity + specificity − 1), the optimal cut-off thresholds were determined. Sensitivity, specificity, and both positive and negative predictive values (NPV) were calculated for these thresholds. Logistic regression analysis was used to examine the relationship between potential risk factors and in-hospital mortality. The results were presented as estimated odds ratios (OR) with 95% confidence intervals (95% CI). A p-value of less than 0.05 was regarded as statistically significant.

## 3. Results

**Fig 1:**
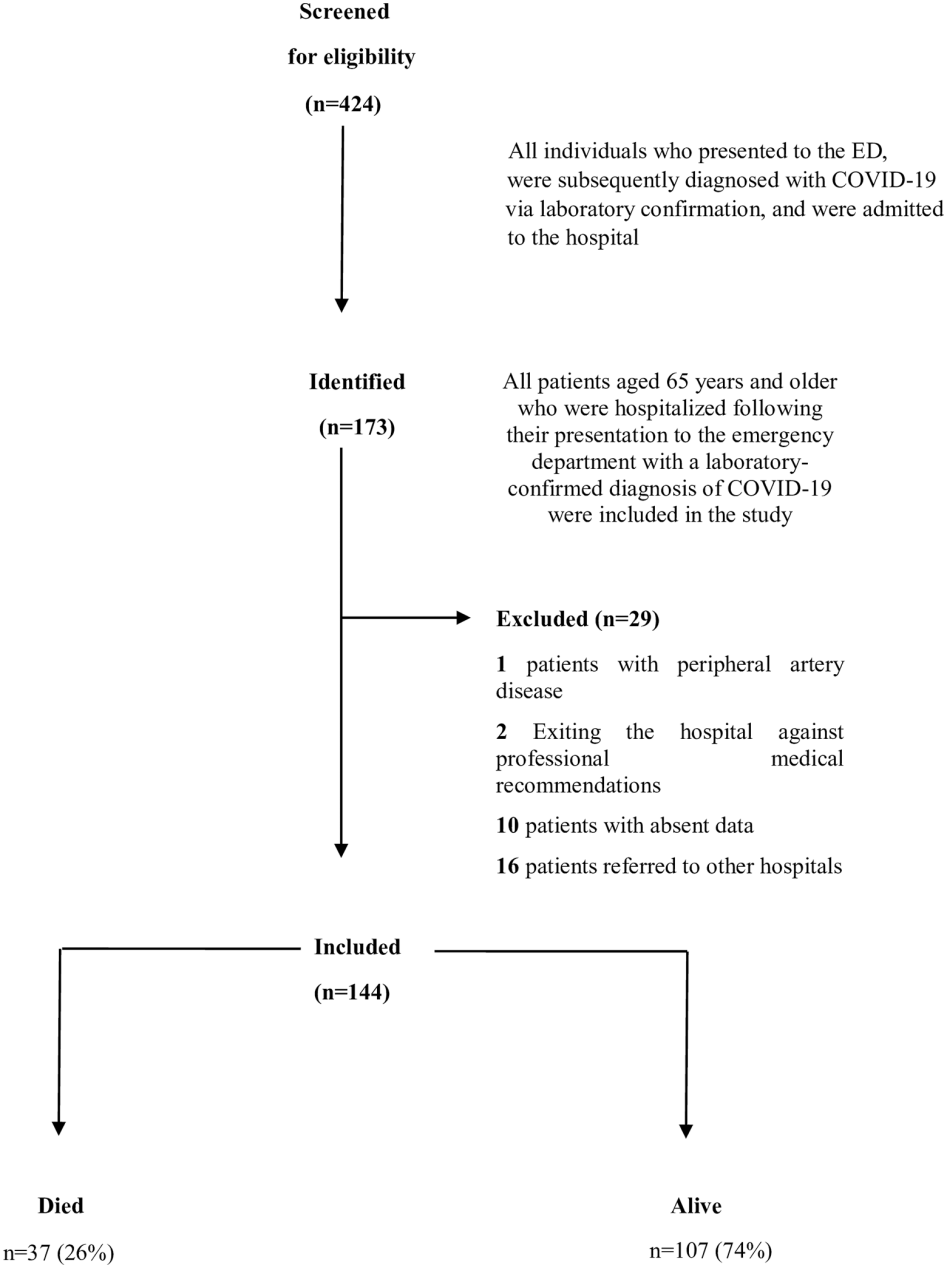
Flow diagram of study cohort.

Between February 15, 2021, and April 15, 2023, a total of 424 patients who were admitted to the ED and subsequently hospitalized with COVID-19 confirmed by RT-PCR were identified. Of these, 173 patients were aged 65 years or older. After applying the predefined exclusion criteria, 29 patients were excluded, and the remaining 144 patients were included in the final analysis (Fig 1).

Of the 144 patients included in the study, 80 (55.6%) were male. Among these patients, 37 (26%) died during hospitalization. Significant differences were observed between the deceased and surviving groups in terms of respiratory rate, oxygen saturation, and PPI. The detailed demographic and clinical characteristics of the patients are presented in Table 1.

**Table 1:** The overview of the demographic and clinical features of patients.

Variable		All patients (n:144)	Survivors (n:107)	Non-survivors (n:37)	*p* value
**Gender, n(%)**	*Male*	80 (55,6 %)	55 (51,4%)	25 (67,6%)	0,088
	*Female*	64 (44,4%)	52 (48,6%)	12(32,4%)	
**Age (years)**		79,15 ± 8,35 80 (65-97)	78,87 ± 8,83 80 (65-97)	79,97 ± 6,79 81 (66-92)	0,464
**LOS (days)**		10,42 ± 13,39 6 (1- 100)	9.27 ± 11,52 6 (1- 100)	13,76 ± 17,51 7 (1- 81)	0,543
**Vital signs**	*Systolic BP (mmHg)*	139,75 ± 29,85 138 (73-237)	141,70 ± 29,84 139 (82-237)	134,11 ± 29,57 133 (73-204)	0,286
	*Diastolic BP (mmHg)*	75,90 ± 15,99 77(37-127)	75,27 ± 15,78 77(37-127)	77,70 ± 16,70 79(44-114)	0,514
	*Pulse (per minute)*	93,82 ± 20,00 93 (40-154)	93,55 ± 19,71 92 (42-154)	94,59 ± 21,06 94 (40-150)	0,687
	*Respiratory rate (per minute)*	21,67 ± 6,11 20 (12-36)	20,73 ± 5,45 20 (32-36)	24,41 ± 7,12 25 (12-36)	**0,006**
	*Saturation (%)*	88,56 ± 9,63 90 (45-100)	89,75 ± 8,91 92 (50-100)	85,14 ± 10,88 86 (45-100)	**0,008**
	*Fever (°C)*	36,67 ± 0,78 36,5 (35,1- 40,2)	36,71 ± 0,88 36,50 (35,1- 40,2)	36,55 ± 0,26 36,50 (36,0- 37,50)	0,727
	*AASI*	55,38 ± 17,81 53,41 (23,38-125,73)	53,84 ± 16,11 52,69 (24,97-125,73)	59,85 ± 21,64 55,43 (23,38-107,14)	0,173
	*MSI*	0,99 ± 0,28 0,95 (0,43-2,03)	0,98 ± 0,25 0,96 (0,43-2,02)	1,02 ± 0,34 0,90 (0,48-2,03)	0,976
	*PPI*	2,86 ± 2,04 2,30 (0,50-9,30)	3,18 ± 2,10 2,80 (0,50-9,30)	1,93 ± 1,51 1,60 (0,50-6,20)	**0,001**

AASI: Age-Adjusted Shock Index, BP: Blood Pressure, Hgb: Haemoglobin, LOS: Length of Stay, Max: Maximum, Min: Minimum, MSI: Modified Shock Index, PPI:Peripheral Perfusion Index, WBC: White Blood Cell

* Comparisons were conducted between the survivor group and the non-survivor group.

To evaluate the diagnostic performance of selected clinical parameters—PPI, oxygen saturation, and respiratory rate—in relation to in-hospital mortality, a ROC curve analysis was performed. The optimal cutoff values for differentiating mortality were determined as respiratory rate ≥25 breaths/min, oxygen saturation ≤86%, and PPI ≤2.20%.

**Fig 2:**
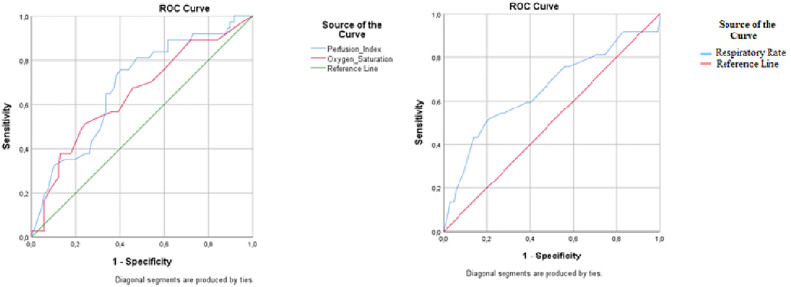
ROC Curve Analysis Showing the Relationship Between Survivors vs Non-survivors. Receiver operating characteristic curves for the Perfusion Index and Oxygen Saturation (**A**); the Respiratory Rate (blue line) (**B**) in relation to in-hospital mortality in COVID- 19 patients.

Among these parameters, PPI demonstrated the highest sensitivity (75.7%) and NPV (87.7%), indicating its utility in identifying patients at lower risk. In contrast, oxygen saturation showed the highest specificity (79.4%) and PPV (46.3%), suggesting that it may be more effective in identifying high-risk individuals. PPI also exhibited the highest area under the curve (AUC = 0.684), whereas oxygen saturation had the lowest (AUC = 0.647) among the analyzed parameters. Detailed results of the ROC curve analysis are presented in Table 2 and Fig 2.

**Table 2:** Diagnostic Performance of Parameters in Differentiating Survivors and Non-survivors.

	AUC (95% CI)	Sensitivity (%)	Specificity (%)	PPV (%)	NPV (%)	*p* value
**Respiratory Rate ≥ 25 breath/min**	0,652 (0,541-0,763)	51,4	75,7	42,2	81,8	**0,006**
**Oxygen Saturation ≤ 86 %**	0,647 (0,541-0,752)	51,4	79,4	46,3	82,5	**0,008**
**PPI ≤ 2,20 %**	0,684 (0,586-0,781)	75,7	59,8	39,4	87,7	**0,001**

PPI: Peripheral Perfusion Index, PPV: Positive predictive value, NPV: negative predictive value, CI: Confidence Interval, AUC: Area Under Curve.

To determine the independent associations of key clinical parameters, a binary logistic regression analysis was performed. Variables found to be statistically significant in the univariate comparisons and ROC curve analysis—namely, PPI (≤ 2.20), respiratory rate (≥ 25 breaths/min), and oxygen saturation (≤ 86%)—were included in the regression model. In the univariate logistic regression analysis, all three variables were significantly associated with in-hospital mortality. A PPI ≤ 2.20% was associated with a more than fourfold increase in mortality risk (OR = 4.630; 95% CI: 1.990–10.776; p < 0.001). Similarly, a respiratory rate ≥ 25 breaths/min (OR = 4.078; 95% CI: 1.838–9.050; p = 0.001) and oxygen saturation ≤ 86% (OR = 3.288; 95% CI: 1.505–7.185; p = 0.003) were also significantly associated with mortality.

Multivariate logistic regression analysis confirmed that each of these parameters remained significantly associated after adjustment for the others. Specifically, PPI ≤ 2.20 (OR = 4.067; 95% CI: 1.668–9.913; p = 0.002), respiratory rate ≥ 25 breaths/min (OR = 3.533; 95% CI: 1.496–8.344; p = 0.004), and oxygen saturation ≤ 86% (OR = 2.456; 95% CI: 1.049–5.753; p = 0.038) were independently associated with increased in-hospital mortality. Detailed results of the univariate and multivariate logistic regression analyses are presented in Table 3.

**Table 3:** Univariate and Multivariate Logistic Regression Analysis for In-Hospital Mortality in Elderly Patients with COVID-19.

	Univariate Logistic Regression Analysis		Multivariate Logistic Regression Analysis	
	*OR (95% CI)*	*p value*	*OR (95% CI)*	*p value*
PPI ≤2,20 %	4,630 (1,990-10,776)	**<0,001**	4,067 (1,668-9,913)	**0,002**
Respiratory Rate ≥25 breath/min	4,078 (1,838-9,050)	**0,001**	3,533 (1,496-8,344)	**0,004**
Oxygen Saturation ≤ 86 %	3,288 (1,505-7,185)	**0,003**	2,456 (1,049-5,753)	**0,038**

OR: Odds Ratio, CI: Confidence Interval, PPI: Peripheral Perfusion Index

## 4. Discussion

This study investigated the factors associated with in-hospital mortality among patients admitted from the ED with a diagnosis of COVID-19. The overall in-hospital mortality rate in our cohort was 26%, which is comparable to previously reported rates among older adults with COVID-19. Our findings underscore the importance of readily obtainable clinical parameters for early risk stratification and triage of high-risk individuals [6,7].

Previous studies have examined the association between vital signs, their derived indices, and mortality among patients with COVID-19. For instance, Balbay et al. reported that decreased oxygen saturation was significantly associated with higher mortality in elderly patients [8], while Mikami et al. identified elevated respiratory rate as a strong indicator of adverse outcomes [9]. Our findings are consistent with these reports. Both decreased oxygen saturation (≤86%) and increased respiratory rate (≥25 breaths/min) were significantly associated with in-hospital mortality in patients aged 65 and older. These results align well with prior studies that emphasize tachypnea as a reliable marker of clinical deterioration in respiratory infections [10]. Elevated respiratory rates are widely recognized as indicators of impaired pulmonary function and increased metabolic demands, both of which are frequently observed in severe COVID-19 cases [11]. Nevertheless, hypoxemia has been widely documented as a key determinant of poor outcomes in COVID-19 patients, with decreased oxygen saturation serving as a surrogate for severe lung injury and impaired gas exchange [12]. These parameters demonstrated strong diagnostic performance in ROC analysis and remained independently associated with mortality in multivariate logistic regression. These results further support the clinical value of vital signs as early and accessible factors linked to disease severity. Their routine use in ED triage protocols may enhance early risk stratification and improve outcomes in elderly patients presenting to the ED with COVID-19.

Among the clinical parameters evaluated in this study, PPI demonstrated the highest association with mortality, with an AUC of 0.684. At a threshold of ≤2.20, PPI exhibited a high sensitivity (75.7%) and a high NPV (87.7%), indicating its usefulness in identifying patients at lower risk for in-hospital mortality. Logistic regression analysis further confirmed the independent association between low PPI levels and increased mortality risk (univariate OR = 4.630, p < 0.001; multivariate OR = 4.067, p = 0.002). Unlike conventional vital signs, PPI offers insight into microcirculatory perfusion and reflects systemic hemodynamic alterations frequently observed in critically ill patients [4]. Our results are consistent with previous studies by Akdur et al. and Korkut et al., both of which showed that reduced PPI is a significant indicator of poor outcomes in COVID-19 [13,14]. While Korkut et al. identified a PPI cut-off value of <2.2 for assessing disease severity, Akdur et al. proposed a lower threshold (PPI <1.5) associated with 14- and 90-day mortality across broader age groups. The slightly higher threshold observed in our study may be attributed to our exclusive focus on patients aged ≥65 and on in-hospital mortality. Taken together, these findings support the incorporation of PPI into early risk assessment protocols for elderly patients with COVID-19. Given its non-invasive nature and ease of measurement, PPI could complement traditional vital signs in guiding triage and management decisions.

This study has several strengths. By focusing on easily accessible and non-invasive parameters, it provides practical insights for ED management, particularly during pandemics when resources are limited. The use of ROC curve analysis to determine optimal thresholds adds statistical rigor to the findings. However, certain limitations must be acknowledged. The retrospective design and single-center nature of the study may restrict its generalizability. Moreover, the exclusion of patients with incomplete data or those transferred to other facilities may have introduced selection bias. Additionally, the study did not evaluate longitudinal changes in these parameters, which might have provided deeper insights into their clinical dynamics over time. Furthermore, due to the retrospective design of our study, systematically collected comorbidity data were not consistently available for all patients in the ED medical record system. Therefore, to avoid the risk of bias arising from heterogeneous and incomplete data, comorbidity information was not included in the analysis. Although the calculated minimum sample size was achieved, the relatively limited cohort size restricts the robustness of predictive modeling. Therefore, the results should be interpreted as associations rather than predictive capabilities.

Future research should aim to validate these findings through multicenter prospective studies with larger cohorts. Integrating advanced imaging techniques and biomarker profiling with the evaluated parameters could further enhance the accuracy and clinical applicability of these parameters, as highlighted by recent systematic reviews on clinical markers in COVID-19. In particular, the strong association of PPI with mortality is increasingly evident in the literature, underscoring its potential role as a practical component of clinical decision-making processes.

## 5. Conclusion

This study highlights respiratory rate, PPI, and oxygen saturation as critical clinical parameters associated with in-hospital mortality in patients aged 65 years and older with COVID-19. However, low PPI emerged as the most significant factor linked to mortality. In healthcare settings, reduced PPI may support patient triage as effectively as conventional vital signs. In light of emerging evidence, low PPI—similar to hypotension and hypoxia —could become an established parameter for assessing disease severity in the ED across diverse clinical conditions.
